# Qualitative and quantitative vibrational spectroscopic analysis of macronutrients in breast milk

**DOI:** 10.1016/j.saa.2020.118982

**Published:** 2021-02-05

**Authors:** Kārlis Bērziņš, Samuel D.L. Harrison, Claudia Leong, Sara J. Fraser-Miller, Michelle J. Harper, Aly Diana, Rosalind S. Gibson, Lisa A. Houghton, Keith C. Gordon

**Affiliations:** a The Dodd-Walls Centre for Photonic and Quantum Technologies, Department of Chemistry, University of Otago, Dunedin 9016, New Zealand; b Department of Human Nutrition, University of Otago, Dunedin 9016, New Zealand; c Faculty of Medicine, Universitas Padjadjaran, West Java, Indonesia

**Keywords:** Breast milk, Raman spectroscopy, Infrared spectroscopy, Chemometrics, Metadata

## Abstract

Raman and attenuated total reflectance-Fourier transform infrared (ATR-FTIR) spectroscopy were used to analyze 208 breast milk samples as part of a larger research study. Comprehensive qualitative and quantitative analysis was carried out using chemometric methods: principal component analysis (PCA) and partial least squares (PLS) regression. The obtained information on the main macronutrients (protein, fat and carbohydrate) were primarily evaluated in relation to the available metadata of the samples, where study location and respective primary food sources revealed a stronger differentiation in fat composition than its absolute content. The limitations and challenges of using both spectroscopic techniques for the type of analysis are also highlighted.

## Highlights

• Qualitative and quantitative Raman and IR spectroscopic analysis of macronutrients in 208 breast milk samples
• Comprehensive spectral data analysis utilizing chemometric methods with included comparison to the available metadata
• Limitations and challenges of using both spectroscopic techniques for the type of analysis were highlighted and addressed

## Graphical abstract

**Figure:**
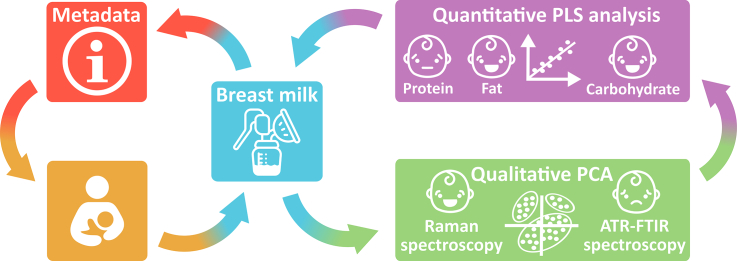

## 1 Introduction

Over the years, vibrational spectroscopic techniques such as Raman and mid-infrared (IR) spectroscopy have been increasingly used for biomedical and biological applications [[1], [2], [3]]. These methods are capable of providing information related to the molecular structure and chemical composition as they probe vibrational motions, which are highly sensitive to changes of these properties [4]. Furthermore, the measurements are typically simple, fast, non-destructive and cost-efficient [5].

Breast milk is a chemically complex system containing protein, lipids, carbohydrates and other biologically active constituents. Being the best source of nutrition during infancy, it is crucial for optimal growth and development of infants, supplying all the essential nutrients that infants need for the first six months of life [6]. It provides unique antibodies and hormones (for example, melatonin), which can help to strengthen the innate immunity system and positively modulate the perinatal health [[7], [8], [9]]. Additionally, unlike standardized infant formula, breast milk composition is dynamic and varies individually, on a daily basis and even during a single breast-feeding session [[10], [11], [12]]. Typically, it consists of around 0.9–1.2% protein, 3.2–3.6% fat and 6.7–7.8% carbohydrate (mainly lactose) with the rest of the matrix comprised of water (>85%) and micronutrients – various minerals and vitamins [[13], [14], [15]]. It is also reported that a significant decline in protein concentration and an increase in fat and lactose concentrations is observed over the first weeks of lactation, largely due to the associated transition from colostrum (first milk) to mature breast milk [16,17]. Depending on the nutrient, the composition of breast milk may be further affected by the maternal obesity and diet [10,18], body stores (available micronutrients and macronutrients) [10,19], personal habits (for example, smoking) [18], mother's age [18], geographical residential area [18], and other factors.

Interestingly, although vibrational spectroscopy has been widely applied for the analysis of range of different milk samples from many species including cow's milk [[20], [21], [22], [23], [24]], goat's milk [25], soymilk [26] and buffalo milk [26], only a few studies have used these techniques for investigating breast milk [[27], [28], [29], [30]]. Additionally, most of the research focus has been limited to qualitative or semi-quantitative analysis. In this study, we utilized both aforementioned techniques (IR and Raman spectroscopy) to thoroughly characterize a number of breast milk samples (208 individual specimens). The application of chemometric methods – principal component analysis (PCA) and partial least squares (PLS) regression allowed for the identification of some correlative trends in regard to available metadata of the samples and to extract reliable quantitative information of at least two of the main macronutrients (fat and carbohydrate).

## 2 Materials and methods

### 2.1 Materials

Breast milk samples were obtained from a study to determine exclusive and partially breastfed infants at 2 months using the deuterium oxide dose-to-mother technique [31]. 212 lactating Indonesian women were recruited. Inclusion criteria for that study were women with no evidence of chronic disease or acute malnutrition who gave birth to a full-term infant (>37-week gestation) with a birth weight ≥ 2500 g, and were currently breastfeeding [31]. In this present study, breast milk samples from 208 lactating mothers were available for macronutrient analysis. Full mature breast milk samples at 2 months postpartum were collected in the morning using a breast pump (Medela AG, Baar, Switzerland), after instructing the mothers to avoid all sources of possible contamination. Aliquots (1 mL) of breast milk were transferred into acid-washed microtubes, frozen on the day of collection (June 2017 to January 2018), and stored at −80 °C prior to analysis, where the samples were allowed to warm up to the ambient temperature (~20 °C). Metadata on gender of the infant, maternal age, median income ranking, food sources, and study location were obtained using an interviewer-administered questionnaire. Maternal body mass index (BMI; kg/m^2^) was calculated from maternal weight and height which were measured using calibrated equipment and standardized techniques [32]. Ethical approval was obtained from the Human Research Ethics Committee, Faculty of Medicine, Universitas Padjadjaran, Bandung, Indonesia (05/UN6.C1.3.2/KEPK/PN/2017). Informed written consent was obtained from all participants.

Calibration and test set milk material (Tables S1 and S2, respectively) were reconstituted from commercial and reference materials, PAMS skim milk powder (PAMS Products Ltd., Auckland, New Zealand), SRM 1846 infant formula (Standard Reference Material 1846, National Institute of Standards & Technology (NIST), MD, USA), 455-C-1 skim milk powder, 453-C-1 buttermilk powder (DairyChek, Global Proficiency Independent Test Verification), and 433-C-1 stage 2 cow's milk infant formula (NurtureChek, Global Proficiency Independent Test Verification). Deionized water was used to reconstitute the powders, using a homogenizer (Heidolph DIAX 900, Schwabach, Germany) at speed level 1 to ensure complete blending. It is important to note that both calibration and test samples were also frozen and thawed to more accurately represent the analyzed breast milk samples (i.e., their sample history).

Ten additional, non-participant samples were analyzed using the Kjeldahl method for protein quality control to judge assay performance of utilized vibrational spectroscopic techniques. As before, breast milk was stored at −80 °C and thawed to ambient temperature (~20 °C) prior to the analysis. Sulfuric acid, sodium hydroxide, boric acid, and hydrochloric acid were purchased from commercial sources.

### 2.2 Fat content quantitative analysis with creamatocrit method

Considering the large number of breast milk samples, we elected to use fast and user-friendly gravimetric fat analysis method to allow for at least an explicit comparison with the spectroscopic results. However, it also must be justly noted that while generally accurate, it can sometimes provide overestimated results when compared to the AOAC or ISO/IDF standard reference methods [33,34]. Using the milk sample aliquoted for the study, glass capillary tubes were filled with gentle shaking between each filling to ensure that the milk fat was well mixed. The capillary tubes were placed in the centrifuge and centrifuged for 3 min. The tubes were then measured using the embedded reader (CreamatocritPlus; EKF Diagnostics, Cardiff, UK) [34]. For each sample, measurements were conducted in triplicate.

### 2.3 Assessment of protein content spectroscopic estimation using the Kjeldahl analysis as a reference method

A different set of ten breast milk samples (see above for more details) was used to assess the accuracy of both utilized spectroscopic techniques for the protein estimation. This approach was utilized as only a limited amount of breast milk was available from the original sample set (insufficient for the Kjeldahl method). The analysis was carried out according to AOAC official method guidelines 991.20 [35]. 4.9830 to 5.1235 g of breast milk was digested in 10 mL of H_2_SO_4_, containing one Kjeldahl catalyst tablet and 4–5 anti-bumping granules. The temperature of the digester (Digestion System 12, 1009 Digester, Tecator, Denmark) was set at 150 °C for 30 min, 190 °C for another 30 min and 330 °C for another 2 h. Afterwards, the translucent samples were cooled down to ambient temperature, quantitatively transferred to 50 mL volumetric flasks, diluted with distilled water, and mixed. Distillation was performed using the Kjeltec 1002 system distilling unit (Foss, Hillerød, Denmark) with 50 mL of 32% NaOH added to the distillation tube and 25 mL of 4% boric acid containing 4 drops of methyl red/bromocresol green indicator solution to the receiving flask. The distillate was titrated with 0.1 M HCl solution to the first trace of pink color, and titrant volume was recorded to the nearest 0.05 mL. The protein content (%) was estimated by multiplying the calculated nitrogen content by a nitrogen-to-protein conversion factor of 6.38. The samples, including blanks, were analyzed in triplicate and the average protein values were blank corrected.

### 2.4 Raman spectroscopy

Raman spectra were collected at room temperature (~20 °C) using a home-built system utilizing an excitation source from a 785 nm laser module (Ondax Inc., Monrovia, CA, USA) which was filtered by BragGrate bandpass filters (OptiGrate Corp., Oviedo, FL, USA) to remove amplified spontaneous emission before irradiating the sample with a laser spot size of approximately 500 μm. For the analysis, glass vials containing the milk samples (~0.5–1 mL) were mounted vertically in a custom-made topside sample holder, positioned above a small stirrer plate, and contents were thoroughly mixed during the data acquisition to ensure their homogeneity. It is also important to note that the vertical position of the glass vials was adjusted in a way that would eliminate an interfering Raman signal from the Teflon coated magnetic stirrer bar. The backscattered light from the sample was collected and filtered through a set of volume Bragg gratings (Ondax Inc., Monrovia, CA, USA) and focused into a LS 785 spectrograph (Princeton Instruments, Trenton, NJ, USA) via a fiber-optic cable. The light was dispersed onto a CCD detector (PIXIS 100 BR CCD, Princeton Instruments, Trenton, NJ, USA) and the data were calibrated using a sulfur, 1,4-bis(2-methylstyryl) benzene (BMB), and a toluene and acetonitrile solvent (1:1) standards. Spectra were collected over the spectral window −360 to 2030 cm^−1^ with 5–7 cm^−1^ resolution. For a typical measurement, each spectrum was averaged from 600 scans with an integration time of 1 s. Due to the prolonged data acquisition time (~10 min), a replicate measurement was only recorded for every five breast milk samples to ensure/validate the robustness of the instrumental setup over time.

### 2.5 Attenuated total reflectance-Fourier transform infrared (ATR-FTIR) spectroscopy

The spectra were recorded at room temperature (~20 °C) on a Bruker VERTEX 70 FTIR spectrometer (Bruker Optics, Ettlingen, Germany) fitted with a GladiATR diamond ATR accessory (Pike Technologies, Madison, WI, USA). Before the analysis, the samples were homogenised using a vortex mixer for 30 s; they were then vigorously shaken and additionally mixed inside a Pasteur pipette before placing a milk droplet on the diamond cell with its position secured using a pressure anvil with a concaved contact tip. Each spectrum was the result of 128 coadded scans over 300 to 4000 cm^−1^ at a 4 cm^−1^ spectral resolution. For each of the breast milk samples, at least three individual replicate measurements were recorded. The spectral dynamics of selected samples were assessed by continuous, single scan measurements over a 60 second time period that were recorded immediately after placing the droplet on the ATR diamond cell.

### 2.6 Data pre-processing

The collected spectra were first converted to .csv from .spe or .0 (OPUS) file format using SpectraGryph 1.2.14. (Dr. Friedrich Menges) [36]. Varied baseline corrections were applied on different spectral regions using Orange Canvas 3.24.1 spectroscopy module (University of Ljubljana, Ljubljana, Slovenia) [37]. Two individual wavenumber intervals (300 to 1900 and 2500 to 4000 cm^−1^) were selected from the collected IR data with each of them requiring different pre-processing – rubberband and linear baseline correction, respectively. On the other hand, two rubberband baseline corrections were necessary for the Raman data ranges used (750 to 1250 and 1400 to 1800 cm^−1^). The small wavenumber break between the two used data regions was necessary as significant spectral information originating from the sample holder/glass vial was identified as interfering with the analysis. Herein, the rubberband correction refers to a method that determines support points by finding the convex hull of each spectrum, which are connected with linear or spline-like baselines. Scale and scattering variation were treated with a standard normal variate (SNV) correction in The Unscrambler X 10.4 (CAMO, Oslo, Norway).

### 2.7 Principal component analysis (PCA)

Principal component analysis was performed on the pre-processed data using The Unscrambler X 10.4 (CAMO, Oslo, Norway) software and nonlinear iterative least squares (NIPALS) algorithm with 10,000 maximum iterations. The PCA model was evaluated by the means of full cross-validation.

### 2.8 Partial least squares (PLS) analysis

In order to quantify the macronutrients in the breast milk samples, a calibration data set comprised of 29 samples was prepared using an assortment of milk powders and procured reference standards (see Section 2.1 above). This approach allowed us to cover a broad and varied concentration range for protein (0.89–12.60 g/100 g), fat (0.09–13.55 g/100 g) and carbohydrate (5.15–28.60 g/100 g) content in calibration samples. Full information on this dataset is available in the supplementary information (Table S1). Individual PLS regression models were constructed using The Unscrambler X 10.4 (CAMO, Oslo, Norway) software. Raman and IR data were collected and pre-processed using the same procedures as described above. Five individual spectra were recorded for each sample and full (systematic) cross validation was calculated. Additionally, 10 samples which constituted an external, independent test set (Table S2), was used to assess the performance of the obtained calibration PLS models.

## 3 Results and discussion

### 3.1 Overview of the sample metadata

A comprehensive metadata was available for the analyzed breast milk samples that is briefly summarized in this section. In total, information from 10 main categories (for example, anthropology and body composition, dietary, household and empowerment) with more than 300 subcategories was accessible. However, considering the substantial size of this data, only specific attributes were selected to be correlated to the spectroscopic analysis, the rational selection of which was mostly based on their impact already described in the literature [10,18,19]. These included maternal age, study location, gender of the infant, parity, median income ranking and mother's body mass index category (specific characteristics are presented in Fig. S1).

### 3.2 Exploratory qualitative analysis

**Fig. 1:**
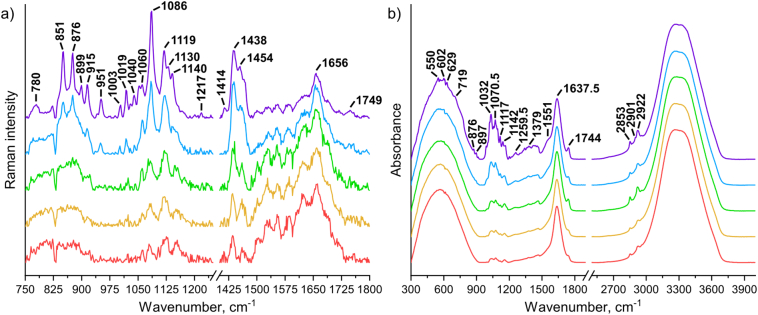
Representative (a) Raman and (b) ATR-FTIR pre-processed spectra for selected calibration samples with progressively increasing protein (1.00 to 5.55 g/100 g), fat (2.44 to 13.55 g/100 g) and carbohydrate (5.15 to 28.60 g/100 g) content (from red to purple graph color).

This study posed a challenge for developing robust data acquisition and processing methodology due to the complex nature of the breast milk samples. Typical Raman and IR pre-processed spectra for range of different calibration samples are shown in Fig. 1. These data also closely mimicked different breast milk samples (Fig. S2). To prevent the potential loss of sensitive spectral information, we opted to only apply linear/rubberband baseline and SNV correction without subtracting interfering signals from the glass vial (most notably, broad underlying features in the range of ~150–200, ~300–500 and ~1000–2000 cm^−1^ in the Raman data; individual spectrum shown in Fig. S3) or water (single or cumulative features at ~570, ~1645 and ~3300 cm^−1^ in the IR data).

For Raman spectra, multiple characteristic bands were identified. The presence of protein was best indicated by the relatively weak amide I band at ~1656 cm^−1^. Lipids could be recognized from two typical modes at 1437 and 1455 cm^−1^ related to δ(CH_2_) scissoring. However, it must be noted that these are overlapping bands which are also attributable to other components in the breast milk matrix. On the other hand, carbohydrates (primarily lactose) showed numerous unique Raman signals varying from weak to very strong with the most notable bands observed at 851, 876, 1086 and 1119 cm^−1^. In concentrated samples, particular disaccharide (its monohydrate form) also displayed a number of low-energy phonon modes (<300 cm^−1^; Fig. S4), which were identified based on the previous work from our group [38].

IR data presented equally sophisticated spectral information. For example, distinct amide III (complex displacements), amide II (N—H bend; C—N stretch) and amide I (C=O stretch) vibrations of the peptide bonds can be seen at around 1259.5, 1551 and 1637.5 cm^−1^, respectively. Milk fats showed several unique yet weak IR bands with ester related C=O stretch at 1744 cm^−1^, and –CH_2_ stretch at ~2853 and 2922 cm^−1^. Lastly, carbohydrates once again exhibited an array of signals, particularly in the range of 900 to 1200 cm^−1^. A full list of Raman and IR spectral peaks and their respective assignments (based on studies by McGoverin et al. [39], Balan et al. [21] and De Marchi et al. [24]) are available in the supporting information (Table S3).

Data collected from the breast milk samples were further processed using the principal component analysis (PCA); a valuable data analysis tool highlighted by a variety of different studies, including ones investigating milk or milk based products [21]. This chemometric technique permits a reduction in the number of variables without the concomitant loss of information with the results represented by scores and loadings. As each point is simplified to a single point in a two-dimensional principal component (PC) space, samples with similar PC scores have related spectral features. Accordingly, loading plots highlight the basis upon which the separation in the PC space has been achieved.

Herein, PCA was focused on the Raman data as fat composition for part of the breast milk samples caused some of the lipid-related bands to be artificially increased in the IR spectra. The results also showed that different sample handling during the ATR-FTIR data collection had a direct impact on the procured spectra for these samples. For example, analysis of a freely placed (unconstrained) droplet resulted in a significant spectral variation, likely caused by a phase separation on the interface level as the milk droplet is left static during the ATR-FTIR data collection, an event that could sometimes even be observed visually. Importantly, such behavior could neither be rectified by more vigorous homogenization of the samples nor improved with a reduced measurement time. Thus, we elected to use a pressure anvil with a concaved contact tip, which helped to reduce some of the artificial variance between samples/replicates by restraining the sample size/volume, but only after a careful selection of the experimental setup. It is important to note that in some instances during the preliminary analysis, the use of a specific instrument accessory (for example, flat contact tip) and extent of the applied force amplified the phase separation (Fig. S5), potentially due to the promoted/preferential attachment of the fat globules to the ATR diamond cell. Aforementioned limitations of the particular technique are discussed in more detail for the quantitative analysis.

**Fig. 2:**
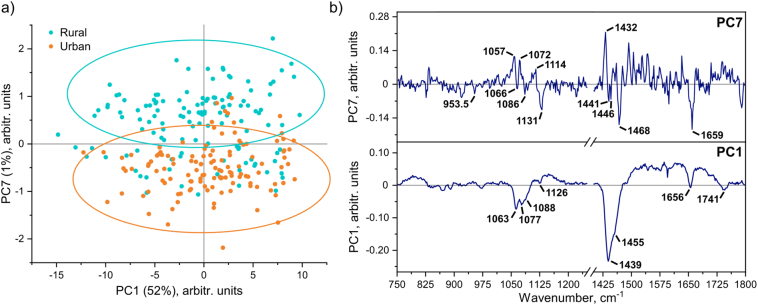
(a) Scores plot of PC1 versus PC7 (with related metadata for the residential areas) and (b) the respective loading plots from PCA of the Raman spectroscopic data for the breast milk samples (including replicate measurements).

**Fig. 3:**
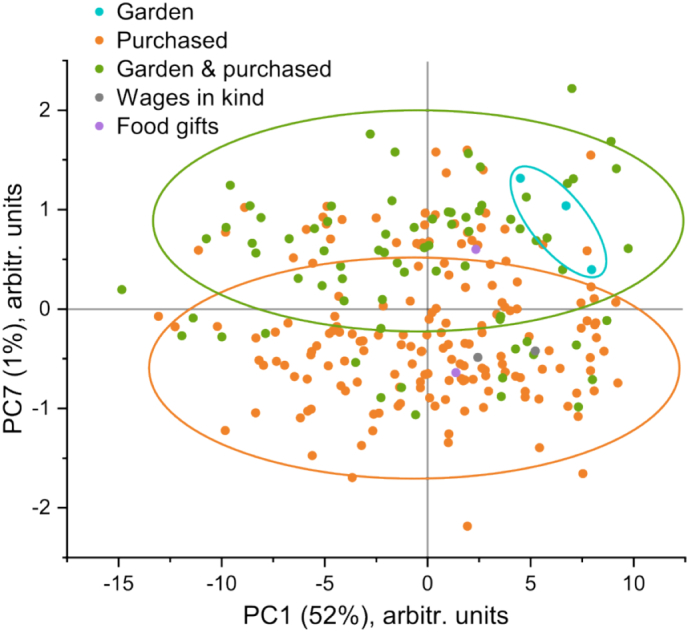
Scores plot of PC1 versus PC7 from PCA of Raman spectroscopic data for the breast milk samples (including replicate measurements) highlighting the associated metadata of primary food sources.

PC1 accounted for around half of the spectral variance within the analyzed data set (52%) and largely resolved intensity based differences for all of the major macronutrients (especially fat), whereas some of the later PCs (PC2 (9%), PC3 (2%), PC4 (2%), PC5 (2%) and PC6 (1%)) appeared to mainly discern some baseline related discrepancies (Fig. S6). This analysis interpretation was also supported by the observed correlation between PC1 score values and determined fat content (described below in more detail), which was absent for the other aforementioned PCs (Fig. S7). Interestingly, PC7 (1%) distinguished a few characteristic bands to fatty acids in the range of 1050–1075, 1090–1120 and 1430–1470 cm^−1^ (Fig. 2b). It has been previously reported that these features in the Raman spectra can be directly linked to the degree of unsaturation and chain length of the particular lipid species [40]. Generally, shorter chain length results in a redshifted peak position, for example, a switch from behenic to myristic fatty acid (22 to 14 carbon chains) causes a peakshift from ~1113 to ~1092 cm^−1^ [40]. Based on these preliminary results, a more detailed study regarding the fatty acid composition of these and other samples (collected at later visits) is currently planned to be carried out. Fig. 2a shows PC1 versus PC7 scores plot, which proved particularly useful to correlate the chemometric analysis to some of the available metadata. Although a number of different attributes (see Section 3.1) were tested, only the study location and simplified dietary information appeared to show definitive trends. More specifically, there was a separation between the breast milk samples provided from the study participants in rural (Sumedang district site) and urban areas (one of the most populated slum subdistricts of Bandung municipality), which directly paralleled the metadata of primary food sources (Fig. 3).

### 3.3 Quantitative analysis

The summary for all of the constructed PLS models are presented in Table 1. In all cases, no more than four factors were necessary for an appropriate and rational PLS regression with the specific spectral features of protein, fat and carbohydrate being highlighted. The main plots for predicted versus reference data points and loadings for the associated factors are also presented in the supplementary information for completeness (Figs. S8–S13). The potential bias of highly leveraged (concentrated) samples was also evaluated. Another set of PLS models were constructed by excluding these samples and their performance was directly compared. Overall, the impact was considered to be negligible as, depending on the number of removed samples, the average absolute difference for predicted fat and carbohydrate values (more reliable parameters; discussed below) was no more than 0.26–0.55 g/100 g.

**Table 1:** Summary of the performance for the PLS regression models created using either Raman or ATR-FTIR spectroscopic data.

	Calibration						Cross validation						Test set			
	Technique	Factors	Slope	Offset	RMSEC	R^2^	Slope	Offset	RMSECV	R^2^		Technique	Slope	Offset	RMSEP	R^2^
Protein (0.89–12.60 g/100 g)	Raman	4	0.98	0.082	0.37	0.98	0.88	0.40	0.59	0.95	Protein (0.71–2.00 g/100 g)	Raman	1.3	0.23	0.74	N/A
	ATR-FTIR	2	0.95	0.20	0.58	0.95	0.94	0.26	0.65	0.94		ATR-FTIR	0.64	1.3	1.0	N/A
Fat (0.09–13.55 g/100 g)	Raman	2	0.96	0.12	0.67	0.96	0.88	0.32	1.1	0.89	Fat (1.74–4.88 g/100 g)	Raman	0.80	0.43	0.38	0.84
	ATR-FTIR	3	0.96	0.11	0.66	0.96	0.90	0.28	0.94	0.92		ATR-FTIR	0.95	−0.0047	0.49	0.75
Carbohydrate (5.15–28.60 g/100 g)	Raman	2	0.97	0.39	1.3	0.97	0.90	1.0	1.8	0.95	Carbohydrate (3.68–10.30 g/100 g)	Raman	0.65	2.8	1.1	0.73
	ATR-FTIR	4	0.99	0.11	0.68	0.991	1.0	0.09	1.3	0.98		ATR-FTIR	0.82	2.1	1.3	0.63

While all of the calibration and cross-validation PLS regression models displayed satisfactory characteristics (slope, offset, RMSE and R^2^ values), the performance of test set models varied. In particular, the protein content determination seemed to be unreliable, irrespective of the utilized analytical technique. It is important to note, however, that the independent test set samples were carefully selected to more closely mimic the more common concentration ranges (Table S2) for each of the macronutrients, and, thus, contained a limited amount of protein (Table 1; 0.71–2.00 g/100 g). Furthermore, specific literature survey revealed that both of the utilized spectroscopic techniques have only been successfully applied to quantify higher levels of protein (>2 g/100 g) [20,41], typical for other types of mammal milk. Hence, it suggests a serious limitation for the applicability of both Raman and ATR-FTIR spectroscopy, which is important to recognize. As mentioned before, fat characteristics in the actual breast milk samples appeared to cause further complications for the analysis of ATR-FTIR spectroscopic data. This aspect affected the performance of all of the developed PLS regression models. According to the fat content values determined from the Raman data, which were also in good agreement to ones obtained with the creamatocrit method (Fig. S14), artificially increased IR bands were more common in fattier milk samples (>6–7 g/100 g). Generally, fat globules in breast milk are larger in size when compared to other types of milk [42], which can facilitate faster phase separation due to the reduced physical stability [43]. Furthermore, with elevated fat content, the volume of fat globules increases rather than their quantity, imposing an even higher risk to the homogeneity of the emulsion [42]. It is also known that freezing/thawing can disrupt fat globule membranes and induce other physical changes that can lead to their reduced stability [44]. All of these factors likely contributed to this observation that was further highlighted by the recorded temporal-spectral dynamics of selected, unstable samples. Overall, the change in lipid-related band intensities for these quasi-homogenous specimens was found to be very fast (<5 s), that also made it difficult to fully characterize the process considering the intrinsic measurement time of a single FTIR scan (~1.5–2 s) and the instrumental delay between them (~1–2 s).

As stated, test set PLS models showed limited reliability for protein estimation. This was reflected by a large number of physically meaningless (negative) predicted values for the actual breast milk samples indicating these were below the detection limit of the model. Additionally, while procured results shared some similarities (Fig. S15), a direct correlation between the Raman and IR data was not observed (Fig. S16). This result coincided with the analysis of the secondary sample set, where Kjeldahl method was used as a reference. Herein, Raman and IR data exhibited similarly poor correlation. Also, in both cases, protein values appeared to be mostly overestimated (Fig. S17), which might indicate that PLS models have not correlated the compositional information with the spectral changes, possibly due to non-related spectral variance. The performance of these PLS models could potentially be improved by including a larger number of calibration samples with more varied protein composition. Although several different standards were used for constructing the calibration curve, it may still be insufficient to fully represent the protein compositional variation within the actual breast milk samples.

**Fig. 4:**
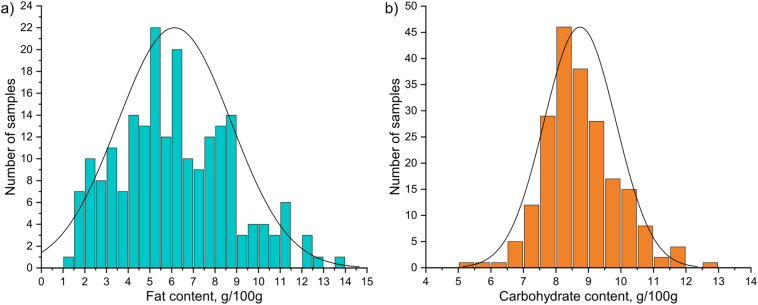
Histograms of the determined average (a) fat and (b) carbohydrate values from the PLS regression models using Raman spectroscopic data.

Fig. 4 shows distributions of the determined average fat and carbohydrate values from the PLS regression models using Raman spectroscopic data. Evidently, there was a much larger disparity in the fat content (ranging from 1.4 to 13.7 g/100 g), which was in line with the literature as it generally shows higher variance when compared to other macronutrients [12]. Fat content is typically higher during the day and evening, and even increases during the breast-feeding session [45,46]. Some studies have speculated this disparity to be also linked to the maternal diet, but an unambiguous relationship has never been fully proved due to number of factors including complexity of the dietary profile and lack of unified handling, storage and analysis procedures [47]. Earlier PCA (Fig. 2, Fig. 3) revealed an apparent correlation of the study location and respective primary food sources to the proportion of different fatty acids in the analyzed breast milk samples. However, in relation to the aforementioned metadata, quantitative values showed insignificant differences (Fig. S18). This might indicate a higher dietary impact on the lipid composition than its total content.

Although no additional reference data were procured for carbohydrates, the results were compared between both spectroscopic techniques. For this analysis, ATR-FTIR data set was edited to remove the spectra exhibiting previously discussed anomalies as unrealistic carbohydrate values were also obtained for these samples. Overall, considerably good correlation was displayed (Fig. S19). The Raman and IR data also shared similar histogram characteristics (including the median value), which were well in line with the literature [48].

### 3.4 Significance

This work highlights some limitations and challenges of using two complimentary techniques – Raman and ATR-FTIR spectroscopy for the analysis of breast milk. This is an important issue as even modern specialized devices such as spectroscopic breast milk analyzers can lack accuracy and precision, and provide varied results [29,49,50]. While both methods have intrinsically different sensitivity to each of the macronutrients (i.e., characteristic vibrational bands), the biggest complications arise from the intricacy of the milk matrix itself. As observed, it is critical to ensure the homogeneity of the samples during the measurements, which may require specific modifications to the experimental setup and may not always be possible (as in the case with ATR-FTIR). Furthermore, protein estimation can be compromised due to the lack of sensitivity in the low concentration range, typical of breast milk. The sheer number of components and presence of overlapping spectral features also render the application of simplistic univariate statistical methods nearly obsolete for the data analysis. Currently, it is hard to assess to what extent these factors have been accounted for in commercial instruments as measurements are typically carried out on immobile samples using a proprietary data collection and analysis software. Nevertheless, we hope that our findings will aid a further refinement of spectroscopic data acquisition protocols and strategies for the particular analysis in the future.

## 4 Conclusions

Raman and ATR-FTIR spectroscopy were used to analyze 208 individual breast milk samples, and qualitative and quantitative information of the main macronutrients (protein, fat and carbohydrate) were extracted with the aid of chemometric methods. From this analysis some parallels between the PCA/PLS results and the available metadata were highlighted. Specifically, participants location of residence (rural or urban) and corresponding information on their primary food sources showed an apparent differentiation in lipid composition, but not its absolute content. Nevertheless, it also presented several serious limitations and drawbacks. For example, both techniques had a limited capability in predicting protein content due to likely sensitivity issues. Additionally, ATR-FTIR spectra for part of the samples (especially ones with a higher fat content) were flawed and displayed lipid bands with elevated intensities due to potential phase separation during the data collection. This aspect affected the performance of all of the derived PLS regression models, and needs to be carefully evaluated if this technique is selected for the breast milk analysis.

## CRediT authorship contribution statement

Kārlis Bērziņš: Conceptualization, Methodology, Analysis and writing.

Samuel D. L. Harrison: Data curation.

Claudia Leong: Methodology, analysis.

Sara J. Fraser-Miller: Conceptualization, Methodology.

Michelle J. Harper: Methodology, analysis.

Aly Diana: Methodology, analysis, Data collection.

Rosalind S. Gibson: Writing - Reviewing and Editing.

Lisa A. Houghton: Writing - Reviewing and Editing.

Keith C. Gordon: Writing - Reviewing and Editing.

## Declaration of competing interest

The authors have no conflicts of interest to declare.

## Appendix A Supplementary data

Supplementary material Image 1
