# Supplementary material for: Qualitative and quantitative vibrational spectroscopic analysis of macronutrients in breast milk
Source: Spectrochim Acta A Mol Biomol Spectrosc. 2021 Feb 5;246:118982. doi: 10.1016/j.saa.2020.118982 (PMC7684643; doi:10.1016/j.saa.2020.118982)
Supplement: Supplementary file 1 — Supplementary material [file mmc1.docx]

**Qualitative and Quantitative Vibrational Spectroscopic Analysis of Macronutrients in Breast Milk**

Kārlis Bērziņš^a^, Samuel D. L. Harrison^a^, Claudia Leong^b^, Sara J. Fraser-Miller^a^, Michelle J. Harper^b^, Aly Diana^b,c^, Rosalind S. Gibson^b^, Lisa A. Houghton^b^, Keith C. Gordon^a*^

^a^The Dodd-Walls Centre for Photonic and Quantum Technologies, Department of Chemistry, University of Otago, Dunedin 9016, New Zealand

^b^Department of Human Nutrition, University of Otago, Dunedin 9016, New Zealand

^c^Faculty of Medicine, Universitas Padjadjaran, West Java, Indonesia

*Corresponding author

**Supporting information**

Table S1. Composition of the calibration samples.

| No. | Powder mass, g | Added water content, g | Component | | | Calculated energy, kcal/100g |
| --- | --- | --- | --- | --- | --- | --- |
|  |  |  | Protein, g/100g | Fat, g/100g | Carbohydrate, g/100g |  |
| *PAMS skim milk powder* | | | | | | |
| 1 | 9.620 | 100.0 | 3.15 | 0.12 | 5.27 | 34.76 |
| 2 | 9.620 | 50.00 | 6.30 | 0.24 | 10.54 | 69.52 |
| 3 | 9.620 | 35.00 | 9.00 | 0.34 | 15.06 | 99.31 |
| 4 | 19.25 | 50.00 | 12.6 | 0.48 | 21.08 | 139.04 |
| *SRM 1846 infant formula* | | | | | | |
| 5 | 4.500 | 50.00 | 1.00 | 2.44 | 5.15 | 46.54 |
| 6 | 4.500 | 45.00 | 1.11 | 2.71 | 5.72 | 51.71 |
| 7 | 4.500 | 40.00 | 1.25 | 3.05 | 6.44 | 58.17 |
| 8 | 4.500 | 35.00 | 1.43 | 3.48 | 7.35 | 66.48 |
| 9 | 4.500 | 30.00 | 1.67 | 4.07 | 8.58 | 77.57 |
| 10 | 4.500 | 25.00 | 2.00 | 4.88 | 10.30 | 93.08 |
| 11 | 4.500 | 15.00 | 3.33 | 8.13 | 17.16 | 155.1 |
| 12 | 4.500 | 11.25 | 4.44 | 10.84 | 22.88 | 206.8 |
| 13 | 4.500 | 9.000 | 5.55 | 13.55 | 28.60 | 258.6 |
| *433-C-1 stage 2 cow’s milk infant formula* | | | | | | |
| 14 | 3.000 | 60.00 | 0.89 | 1.13 | - | - |
| 15 | 4.500 | 45.00 | 1.78 | 2.26 | - | - |
| 16 | 4.500 | 40.00 | 2.00 | 2.54 | - | - |
| 17 | 4.500 | 35.00 | 2.29 | 2.90 | - | - |
| 18 | 4.500 | 30.00 | 2.67 | 3.38 | - | - |
| 19 | 4.500 | 25.00 | 3.20 | 4.06 | - | - |
| *453-C-1 buttermilk powder* | | | | | | |
| 20 | 4.500 | 45.00 | 3.01 | 0.81 | - | - |
| 21 | 4.500 | 40.00 | 3.39 | 0.91 | - | - |
| 22 | 4.500 | 35.00 | 3.87 | 1.04 | - | - |
| 23 | 4.500 | 30.00 | 4.52 | 1.22 | - | - |
| 24 | 4.500 | 25.00 | 5.42 | 1.46 | - | - |
| *455-C-1 skim milk powder* | | | | | | |
| 25 | 4.500 | 45.00 | 3.38 | 0.09 | - | - |
| 26 | 4.500 | 40.00 | 3.80 | 0.10 | - | - |
| 27 | 4.500 | 35.00 | 4.35 | 0.11 | - | - |
| 28 | 4.500 | 30.00 | 5.07 | 0.13 | - | - |
| 29 | 4.500 | 25.00 | 6.09 | 0.15 | - | - |

Table S2. Composition of the test set samples.

| No. | Powder mass, g | Added water content, g | Component | | | Calculated energy, kcal/100g |
| --- | --- | --- | --- | --- | --- | --- |
|  |  |  | Protein, g/100g | Fat, g/100g | Carbohydrate, g/100g |  |
| *SRM 1846 infant formula* | | | | | | |
| 1 | 4.500 | 70.00 | 0.71 | 1.74 | 3.68 | 33.24 |
| 2 | 4.500 | 65.00 | 0.77 | 1.88 | 3.96 | 35.80 |
| 3 | 4.500 | 60.00 | 0.83 | 2.03 | 4.29 | 38.78 |
| 4 | 4.500 | 55.00 | 0.91 | 2.22 | 4.68 | 42.31 |
| 5 | 4.500 | 50.00 | 1.00 | 2.44 | 5.15 | 46.54 |
| 6 | 4.500 | 45.00 | 1.11 | 2.71 | 5.72 | 51.71 |
| 7 | 4.500 | 40.00 | 1.25 | 3.05 | 6.44 | 58.17 |
| 8 | 4.500 | 35.00 | 1.43 | 3.48 | 7.35 | 66.48 |
| 9 | 4.500 | 30.00 | 1.67 | 4.07 | 8.58 | 77.57 |
| 10 | 4.500 | 25.00 | 2.00 | 4.88 | 10.30 | 93.08 |

Table S3. The Raman and IR notable spectra peaks and their respective assignments.

| Raman | | IR | |
| --- | --- | --- | --- |
| Wavenumber, cm^-1^ | Assignment | Wavenumber, cm^-1^ | Assignment |
| 850-920 | ν(C–C), ν(C–O), –CH_3_ rocking | 550-630 | Lactose |
| 1086 | ν(C–C) | 1032 | –CH_2_ bending |
| 1120-1123 | ν(C–C) | 1070.5 | ν(C–O) stretching in lactose |
| 1437-1438 | δ(CH_2_) scissoring | 1259.5 | Amide III |
| 1454-1460 | δ(CH_2_) scissoring | 1551 | Amide II |
| 1656 | Amide I | 1637.5 | Amide I |
| 1749 | ν(C=O) | 1744 | ν(C=O) |
|  |  | 2853 | –CH_2_ stretching |
|  |  | 2901 | –CH_2_ stretching (intrinsic to lactose) |
|  |  | 2922 | –CH_2_ stretching |





Fig. S1. Overview of selected metadata of the breast milk samples. (a) Histogram of the maternal age and bar diagrams of (b) participants location of residence, (c) gender of the infant, (d) parity, (e) wealth index and (f) primary food sources.


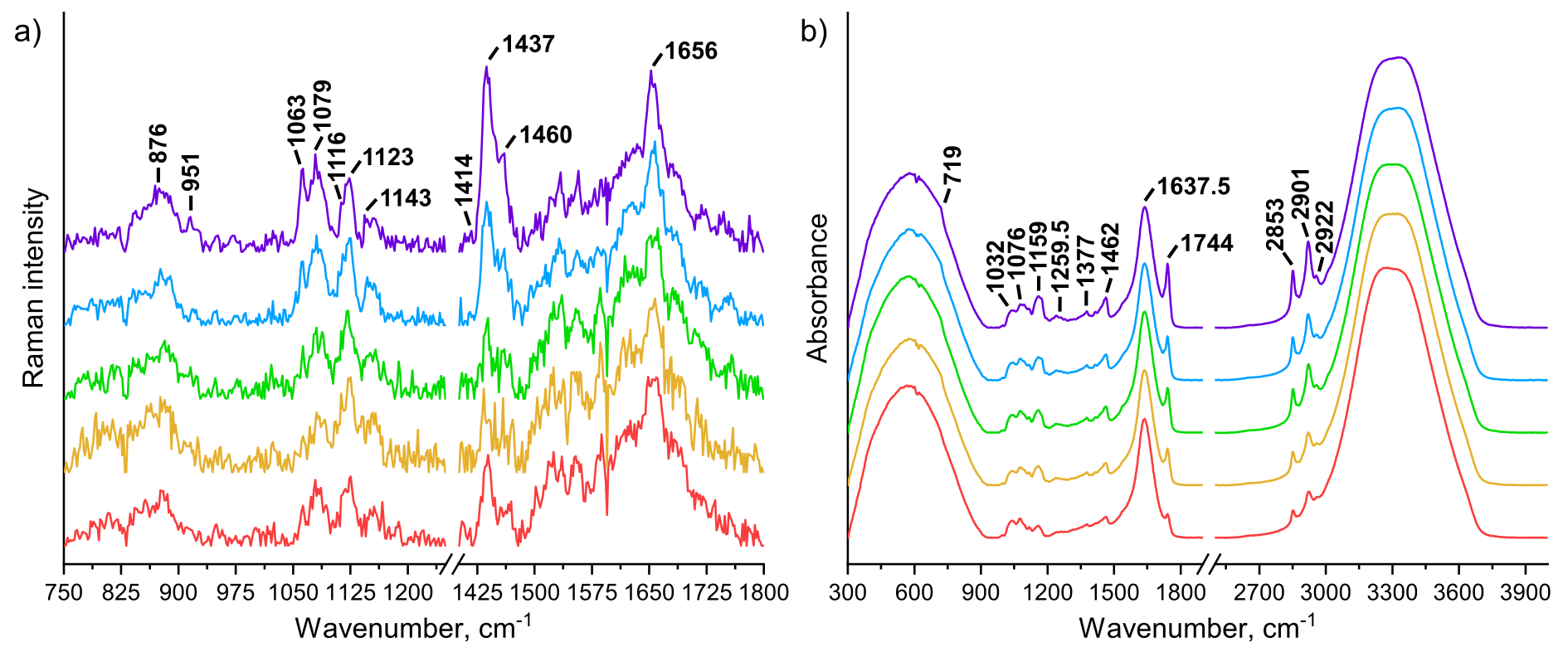


Fig. S2. Representative (a) Raman and (b) ATR-FTIR pre-processed spectra for various breast milk samples.


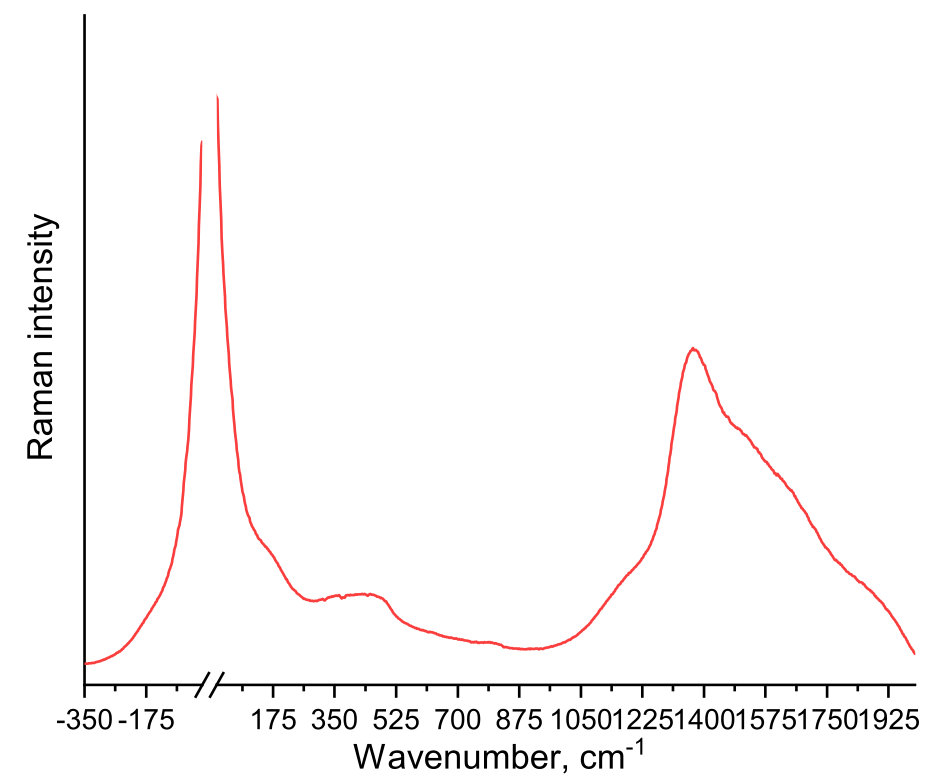


Fig. S3. Representative Raman spectrum of a glass vial/sample holder.


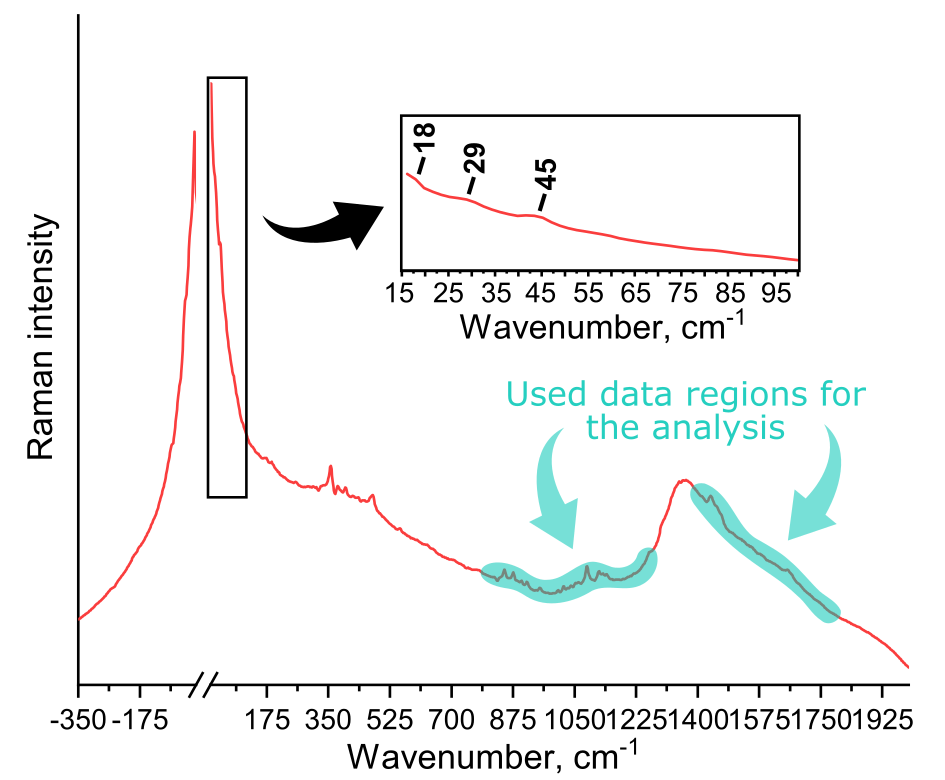


Fig. S4. Representative Raman spectrum of a selected calibration sample highlighting the used data regions for the analysis (in light blue), and low-energy phonon modes associated with the lactose monohydrate.


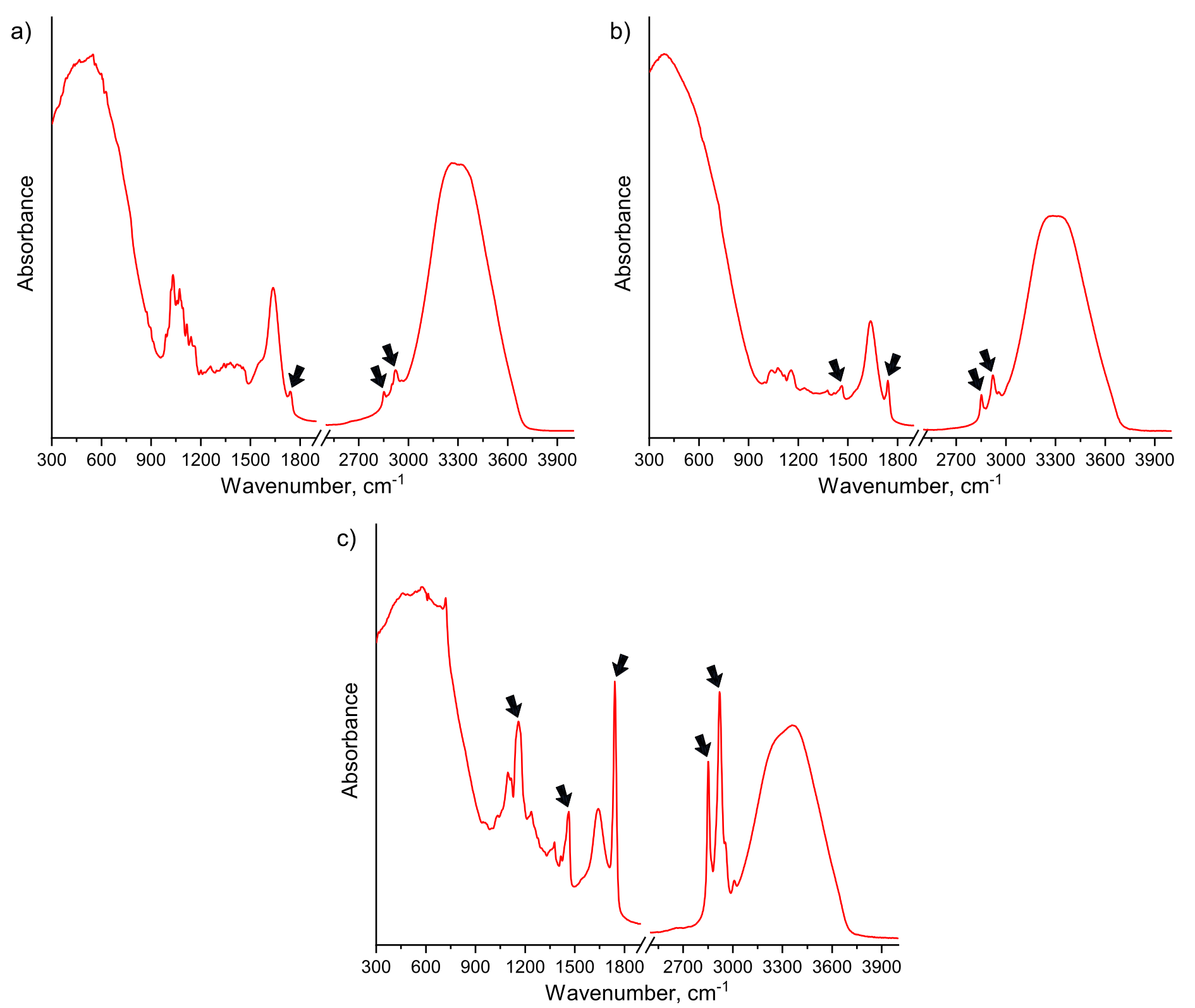


Fig. S5. Representative ATR-FTIR raw spectrum of (a) calibration sample with a high fat concentration of 13.55 g/100g and actual breast milk sample exhibiting some (abnormally) increased bands due to (b) intrinsic phase separation (unconstrained droplet) and (c) amplified phase separation due to applied pressure from the anvil with a flat contact tip.


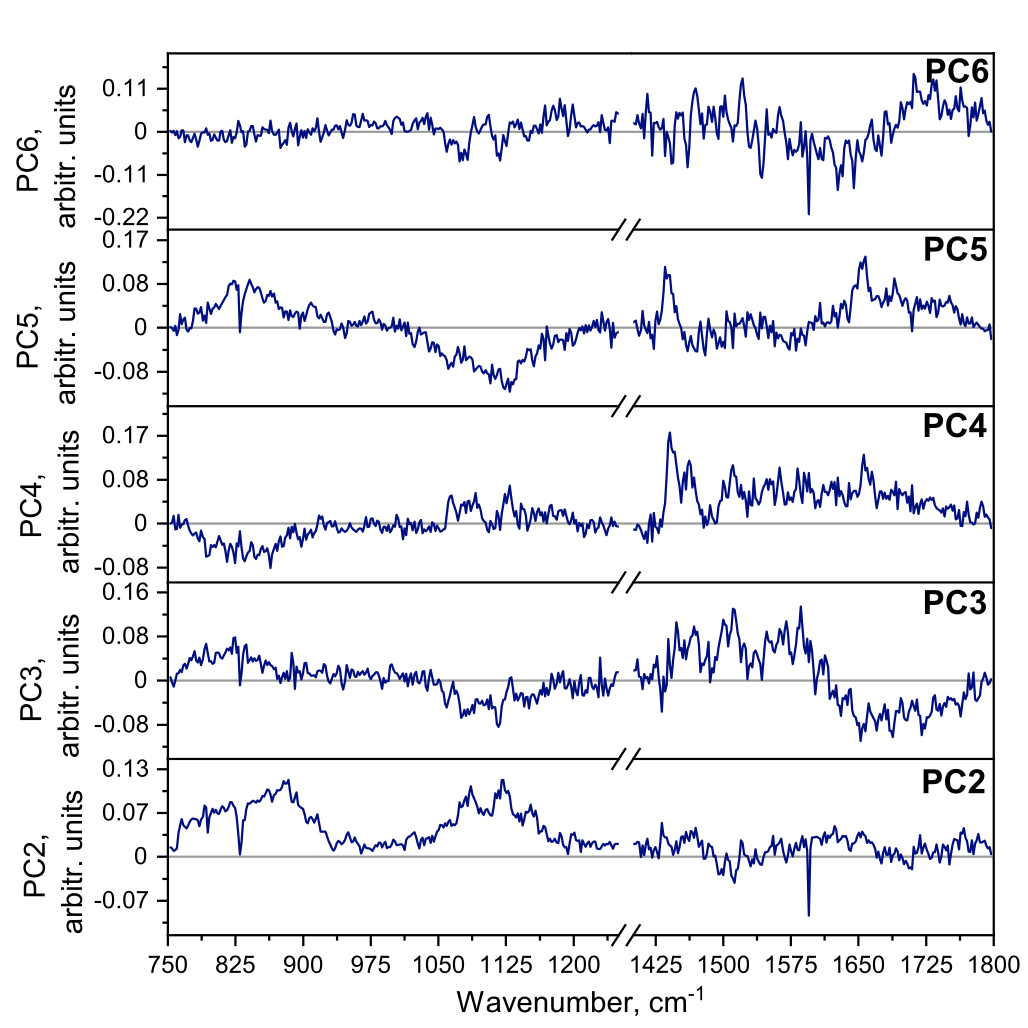


Fig. S6. PC2, PC3, PC4, PC5 and PC6 loadings plots of PCA of the Raman spectroscopic data for the breast milk samples.


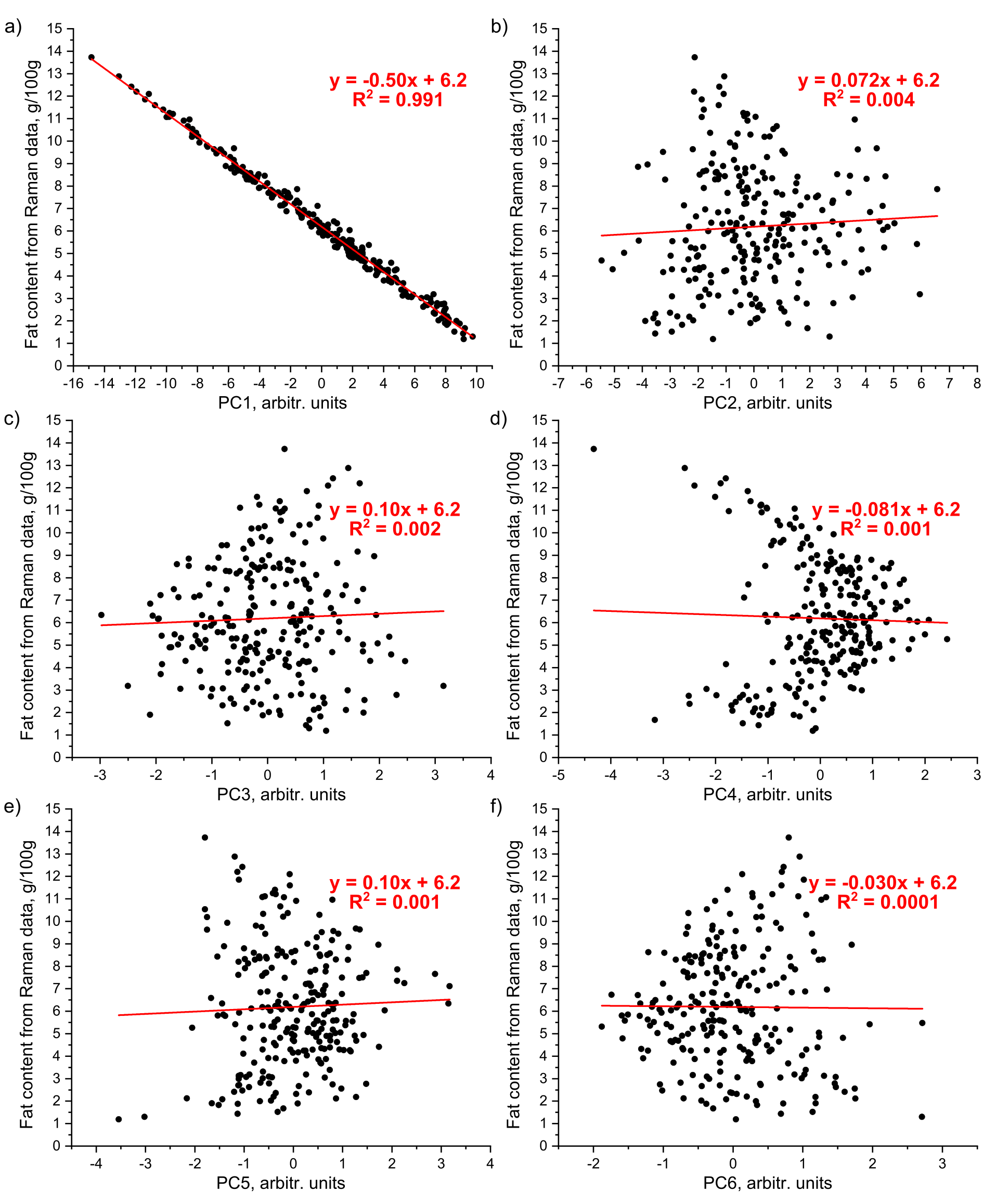


Fig. S7. Comparison of PC1-PC6 score values to determined fat content from the Raman data using PLS regression model.


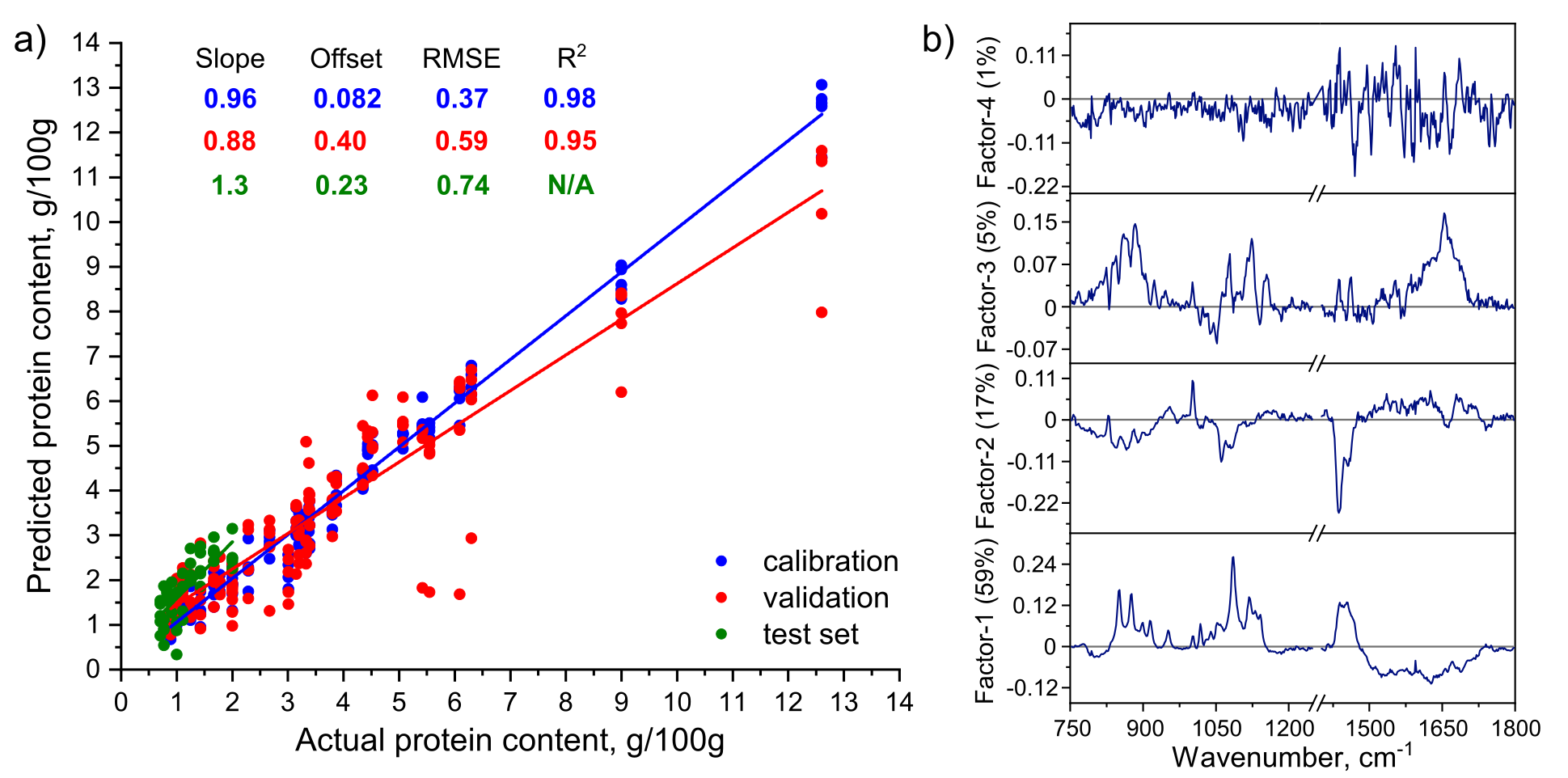


Fig. S8. (a) Calibration, cross-validation and test set data, and (b) loadings plots of factors 1, 2, 3 and 4 of PLS regression model for protein content determination from Raman spectra.


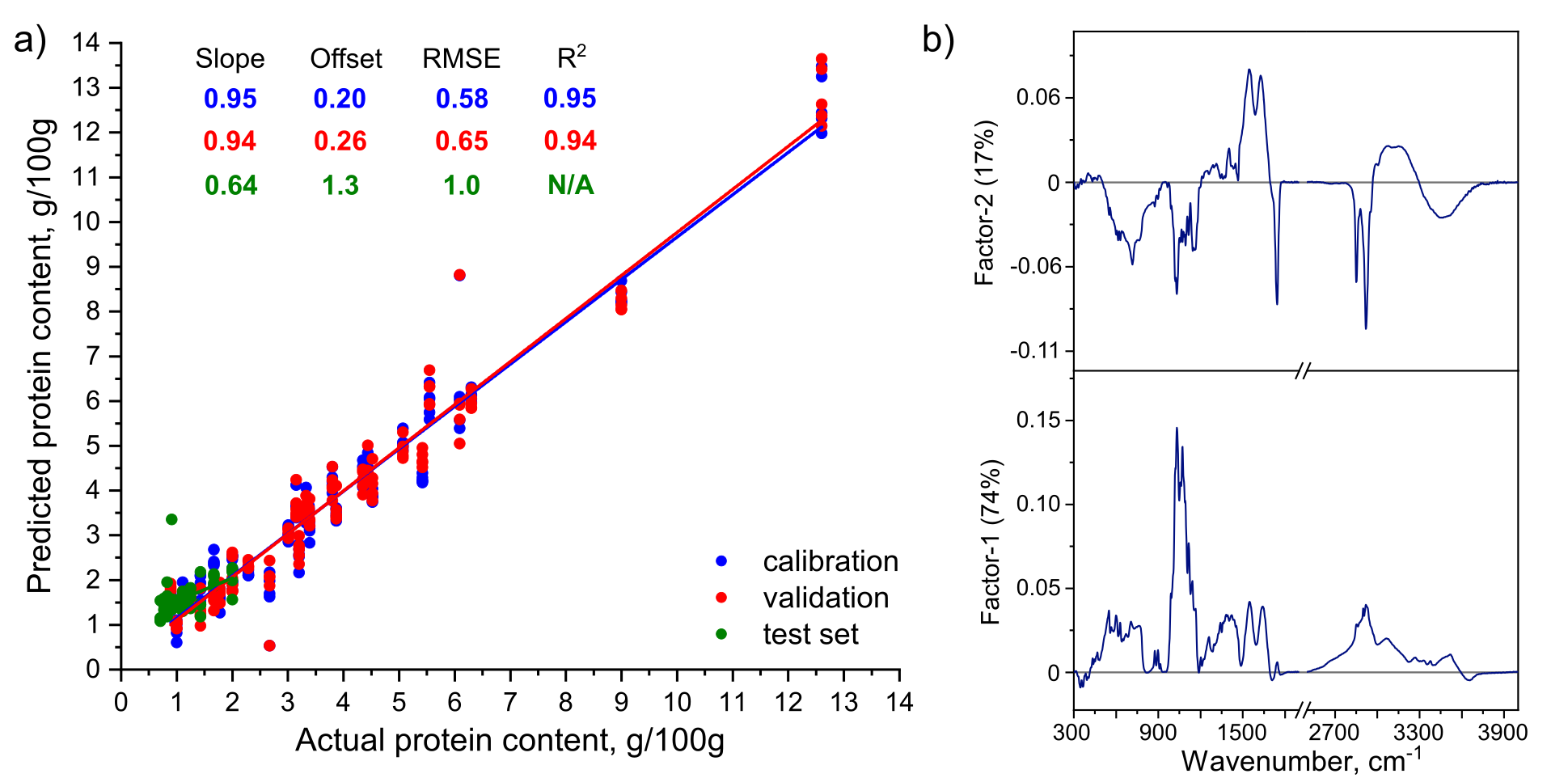


Fig. S9. (a) Calibration, cross-validation and test set data, and (b) loadings plots of factors 1 and 2 of PLS regression model for protein content determination from ATR-FTIR spectra.


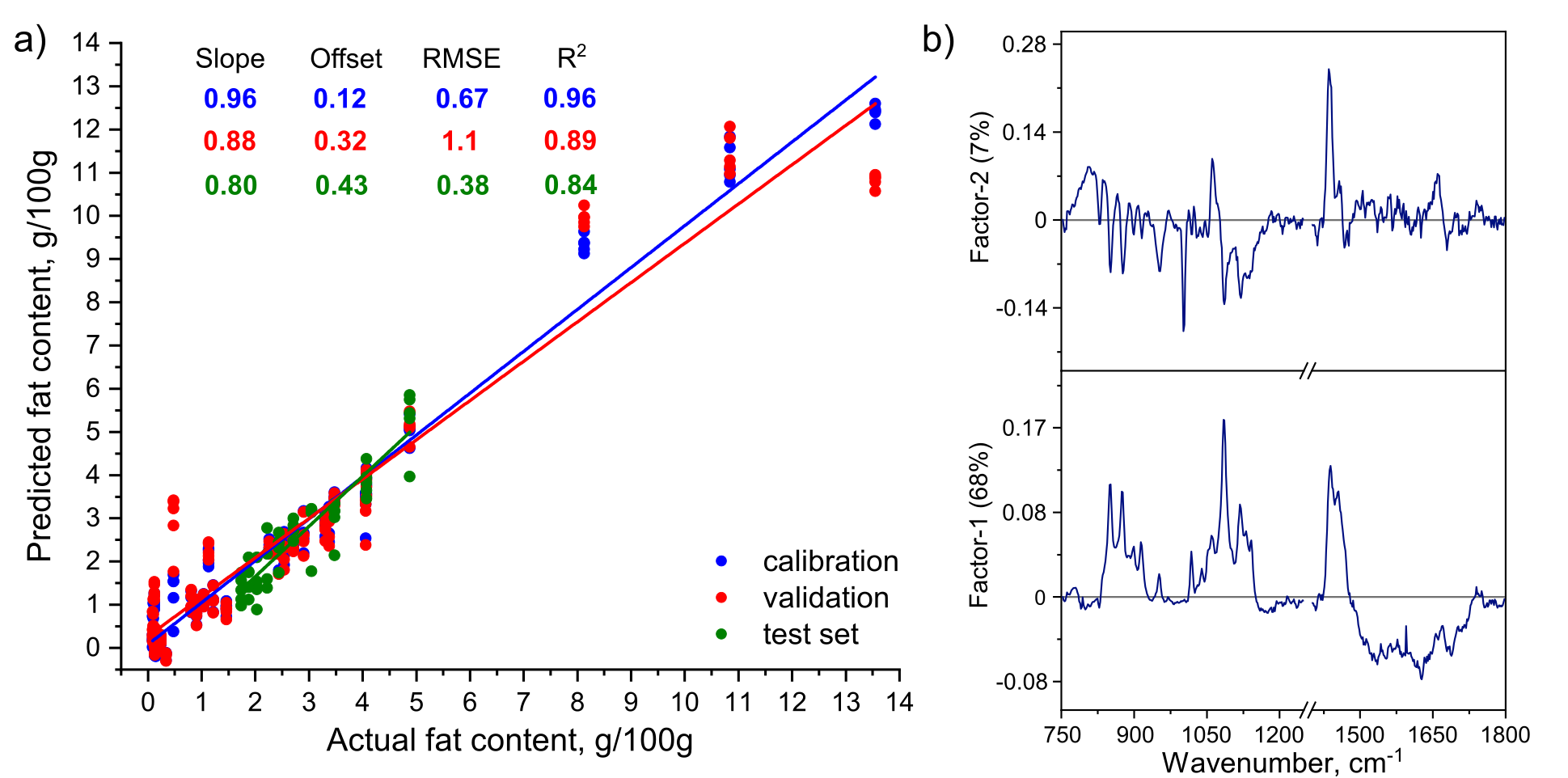


Fig. S10. (a) Calibration, cross-validation and test set data, and (b) loadings plots of factors 1 and 2 of PLS regression model for fat content determination from Raman spectra.


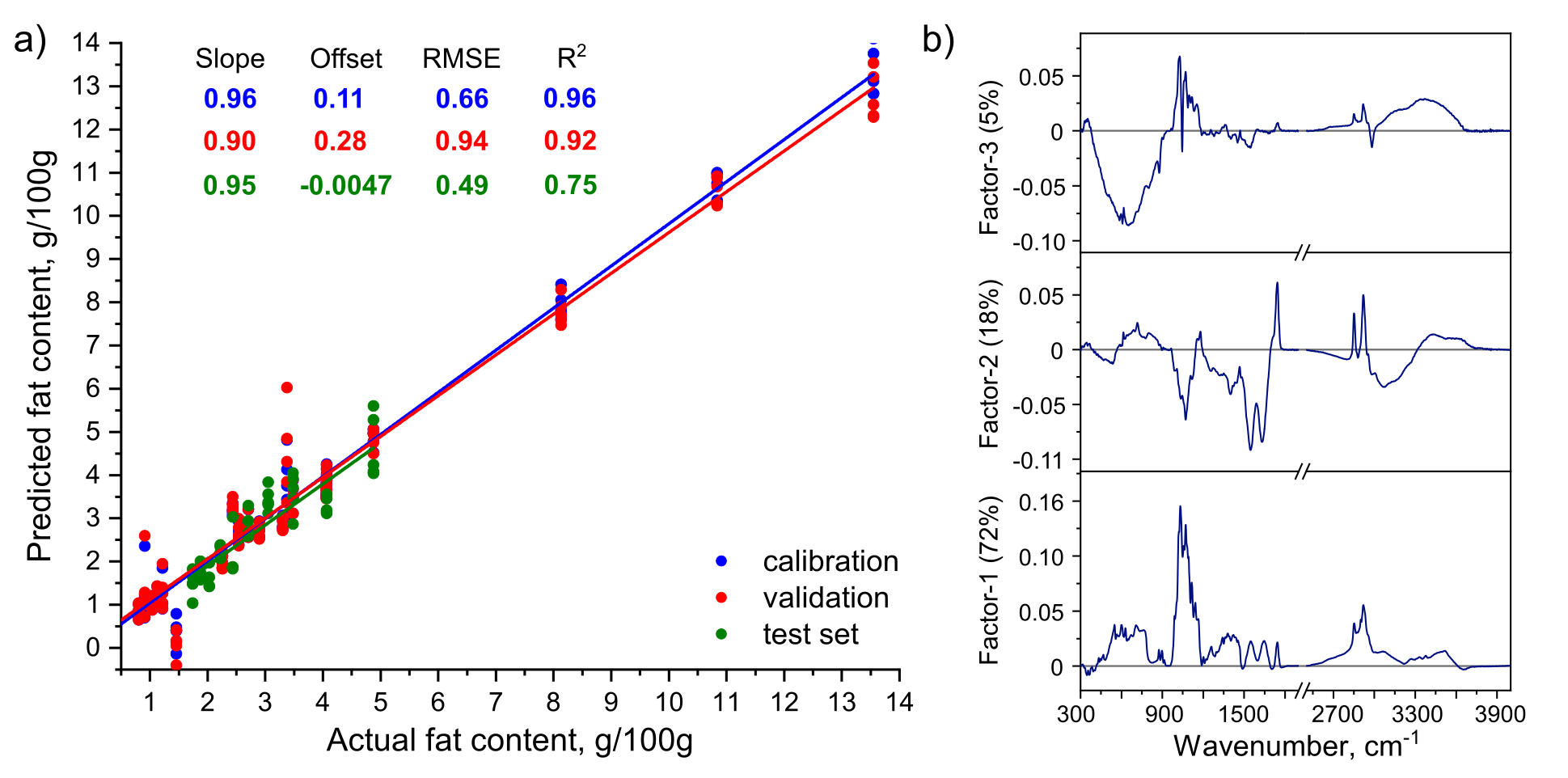


Fig. S11. (a) Calibration, cross-validation and test set data, and (b) loadings plots of factors 1, 2 and 3 of PLS regression model for fat content determination from ATR-FTIR spectra.


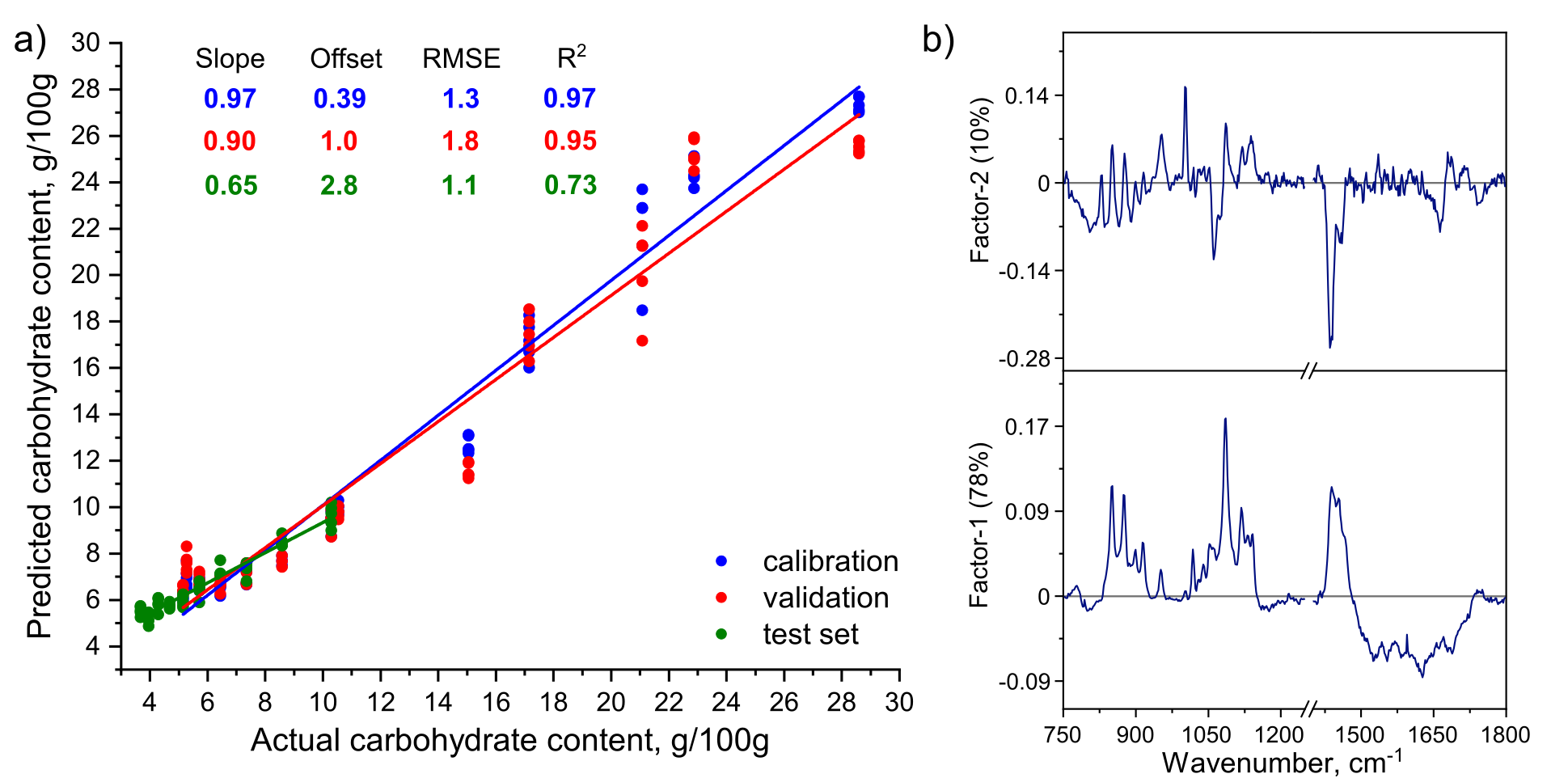


Fig. S12. (a) Calibration, cross-validation and test set data, and (b) loadings plots of factors 1 and 2 of PLS regression model for carbohydrate content determination from Raman spectra.


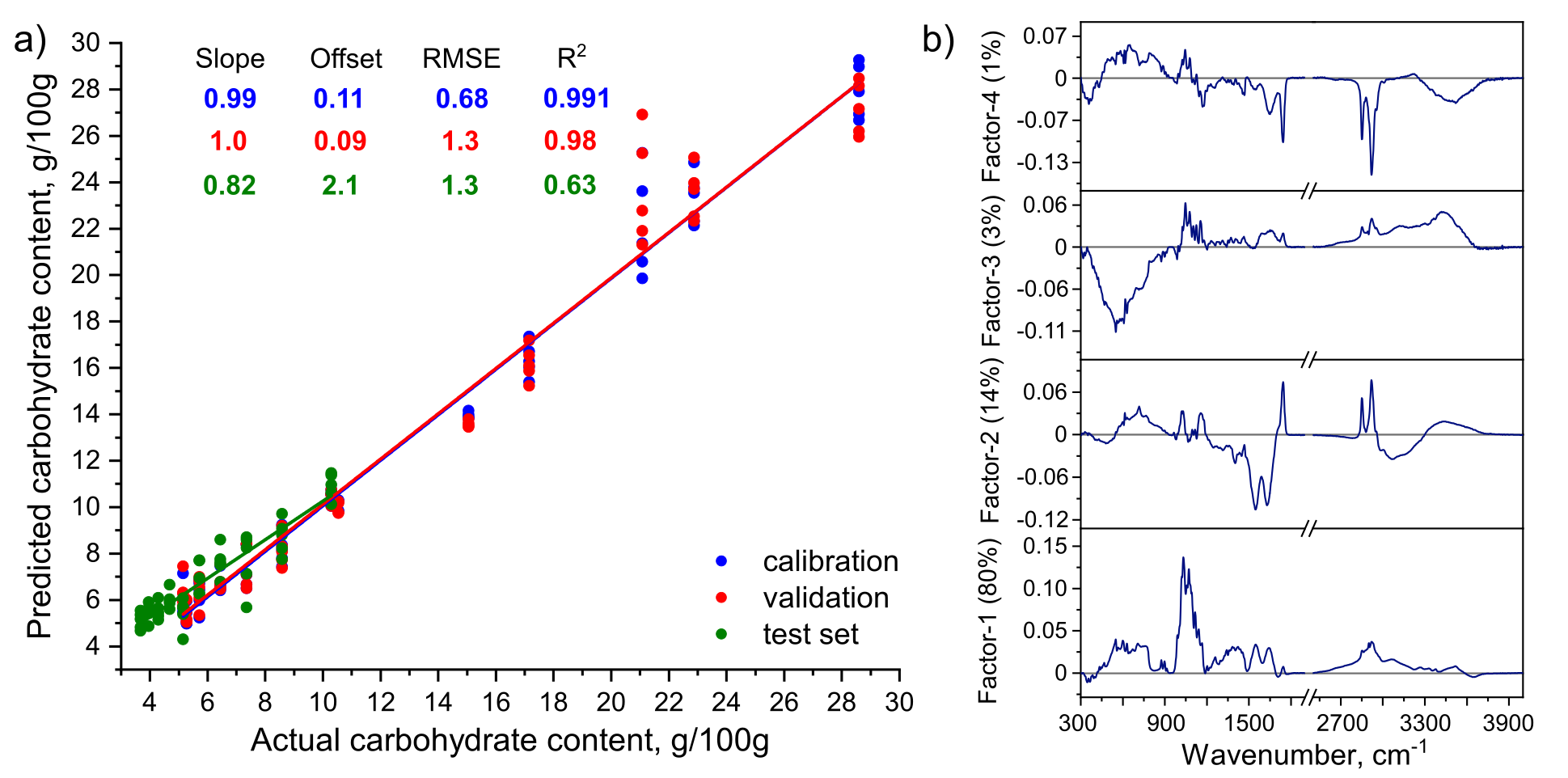


Fig. S13. (a) Calibration, cross-validation and test set data, and (b) loadings plots of factors 1, 2, 3 and 4 of PLS regression model for carbohydrate content determination from ATR-FTIR spectra.


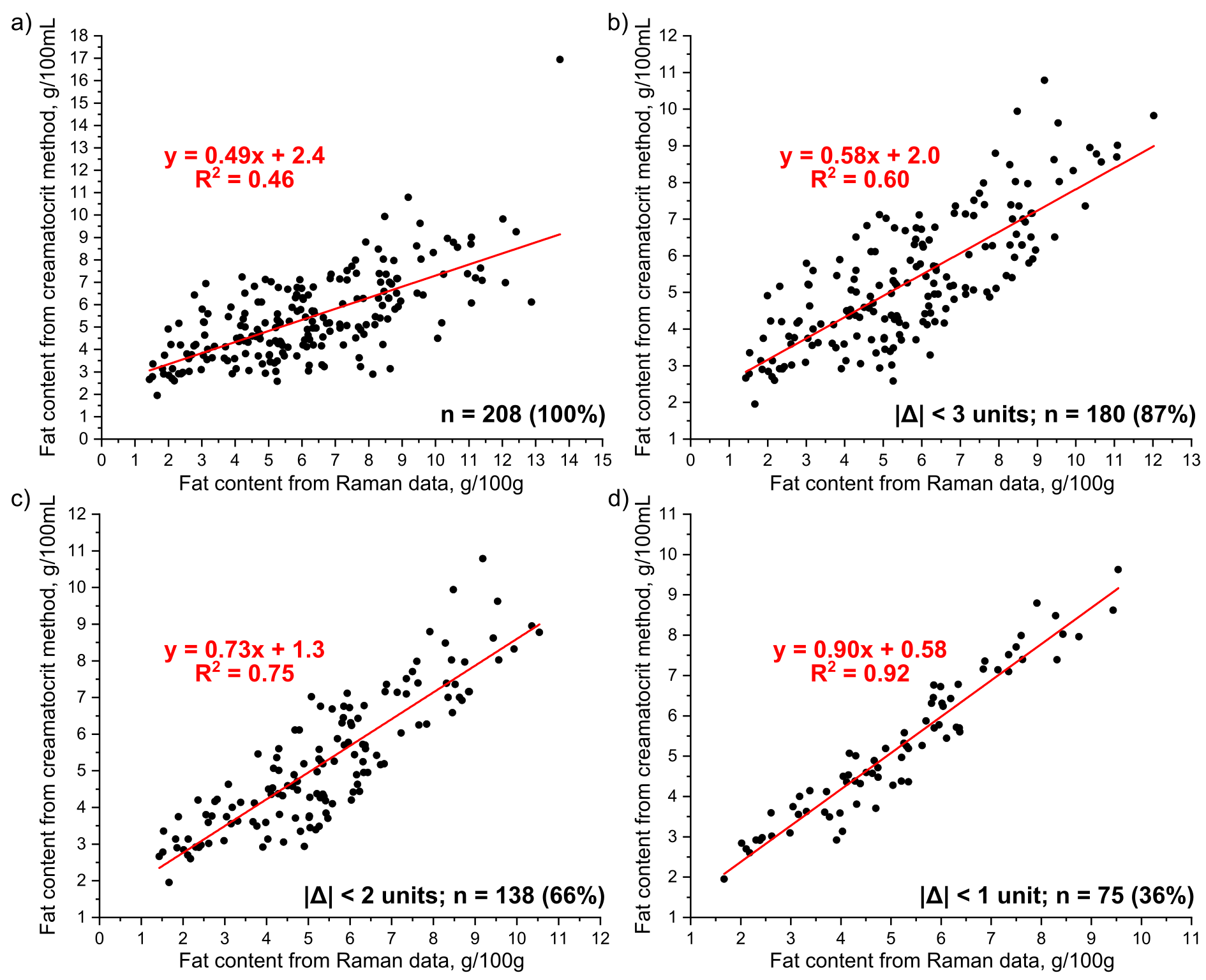


Fig. S14. Comparison of the average determined fat content values from the Raman data and creamatocrit method.


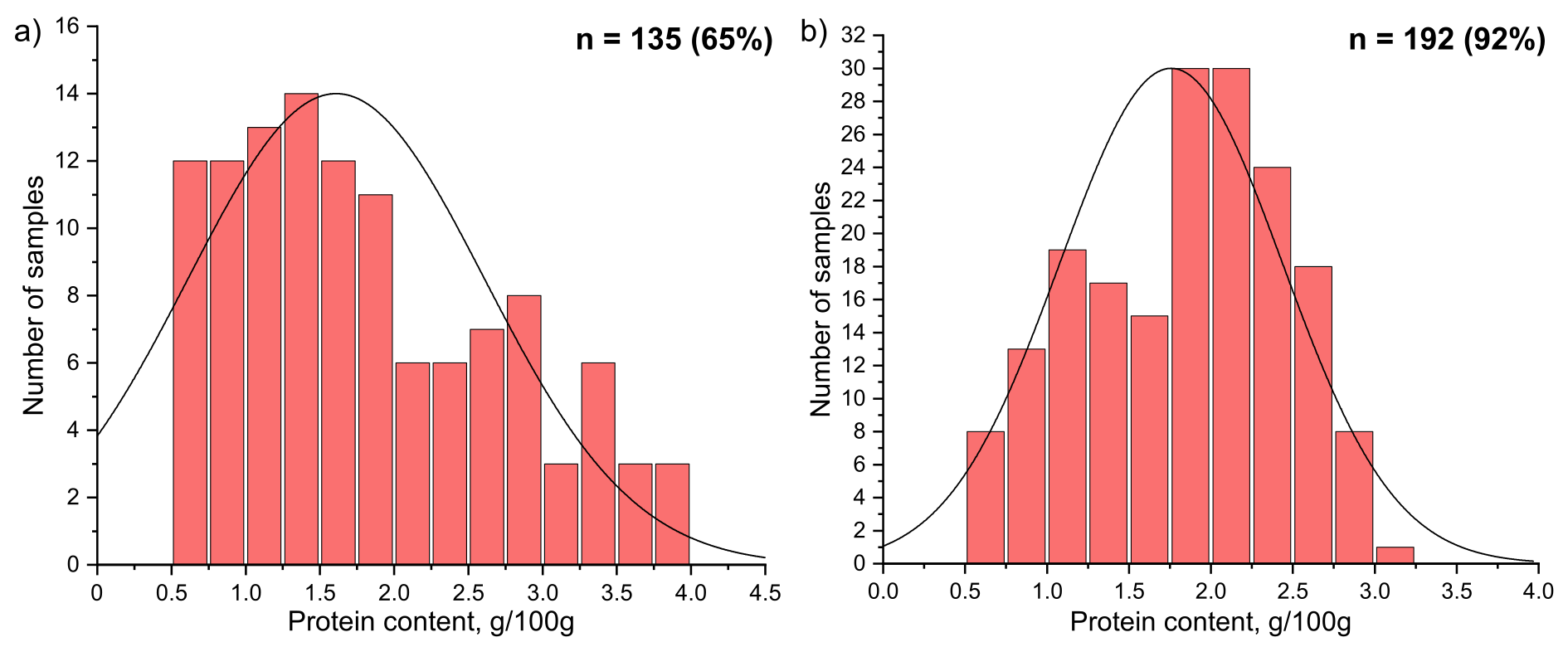
 Fig. S15. Histograms of the determined average protein values from the PLS regression models using (a) Raman and (b) IR spectroscopic data. Only data with meaningful (positive) values are presented.


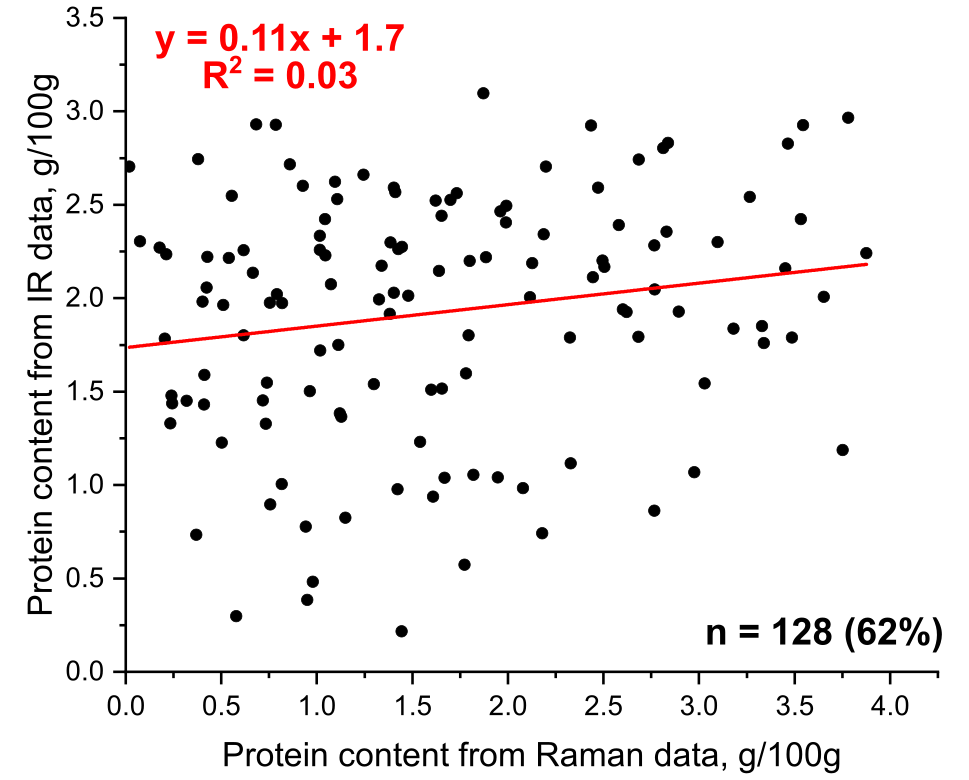


Fig. S16. Comparison of the average determined protein content values from the Raman and ATR-FTIR data. Only physically meaningful (positive) predicted values from both techniques are presented.


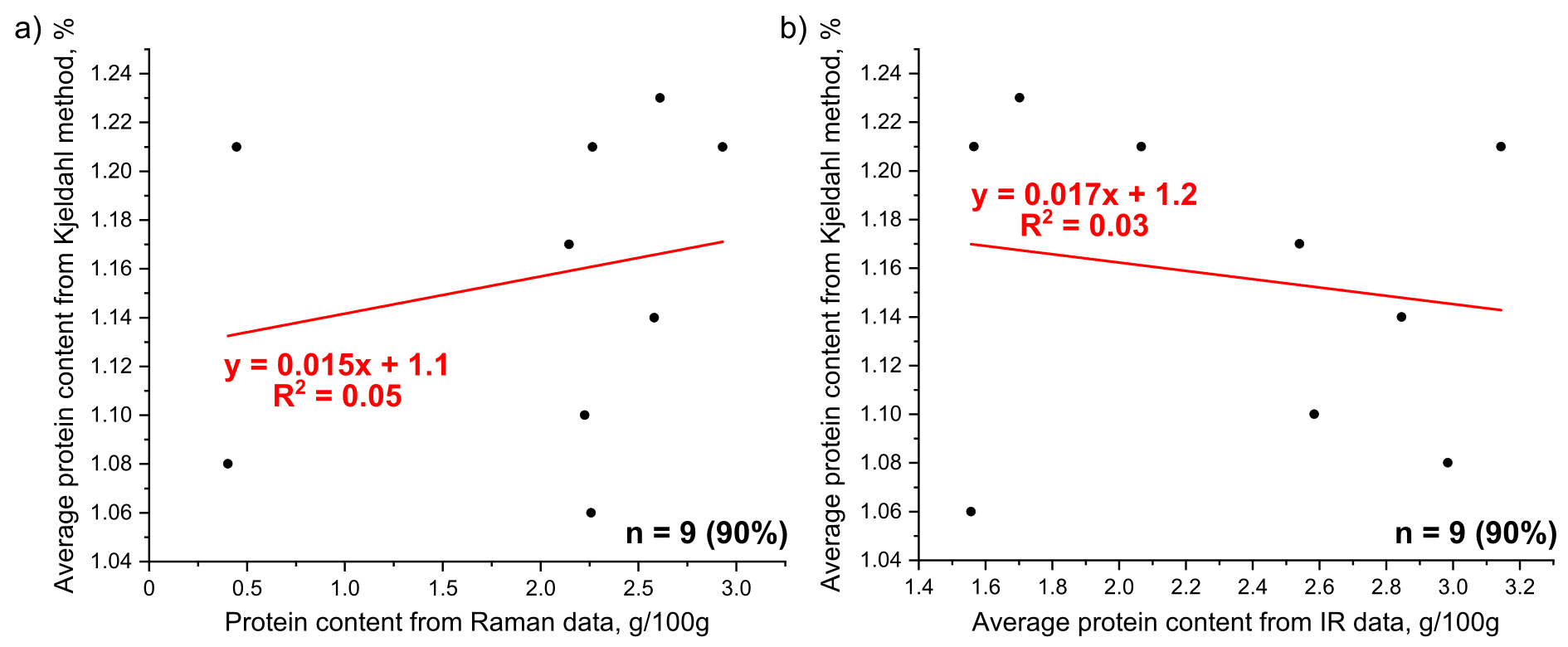


Fig. S17. Comparison of the (average) determined protein content values from the (a) Raman and (b) ATR-FTIR data, and Kjeldahl analysis for the secondary sample set. Only physically meaningful (positive) predicted values from both spectroscopic techniques are presented.


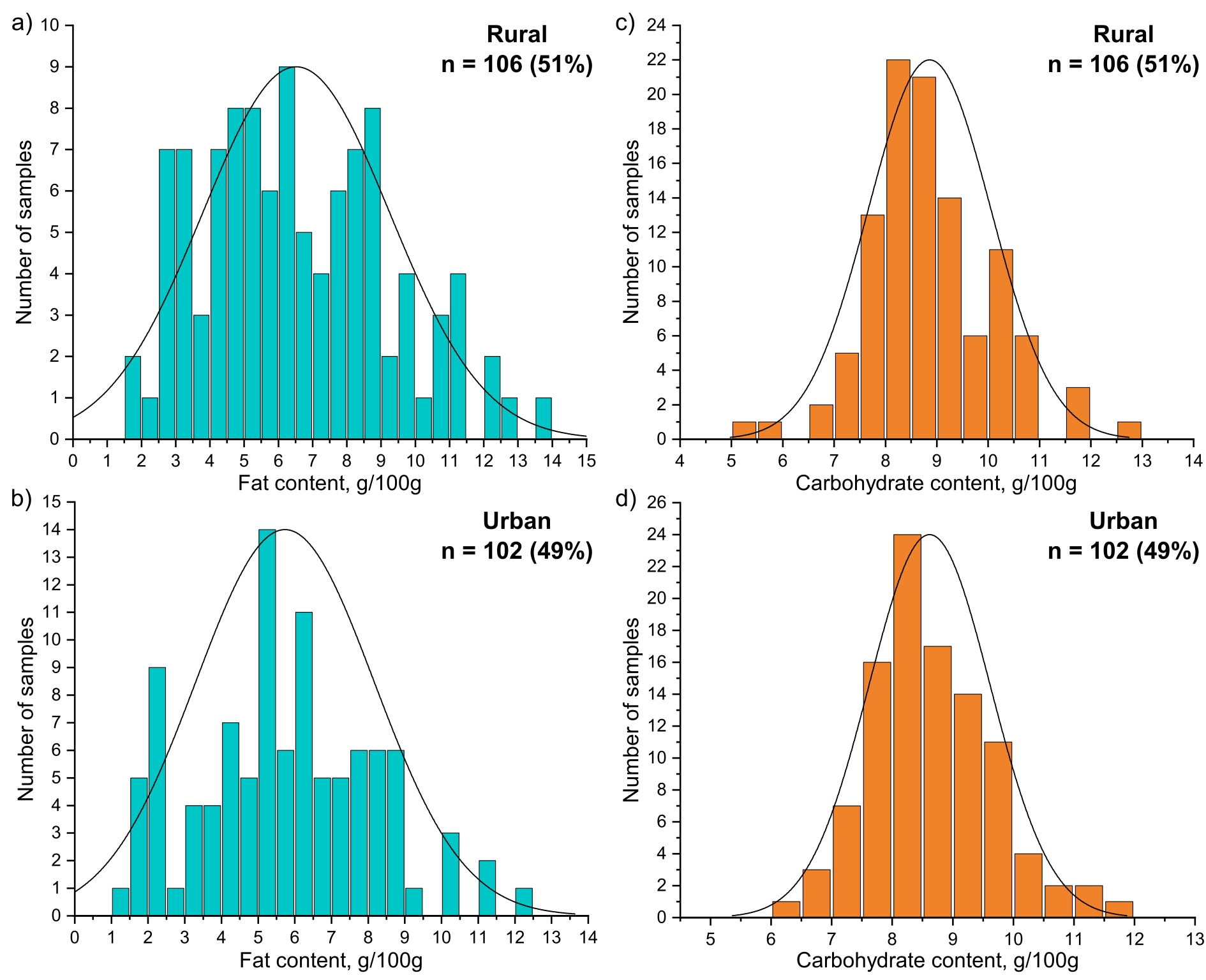


Fig. S18. Histograms of the determined average fat (a-b) and carbohydrate (c-d) values from the PLS regression models using Raman spectroscopic data in relation to the study location metadata (rural and urban areas).


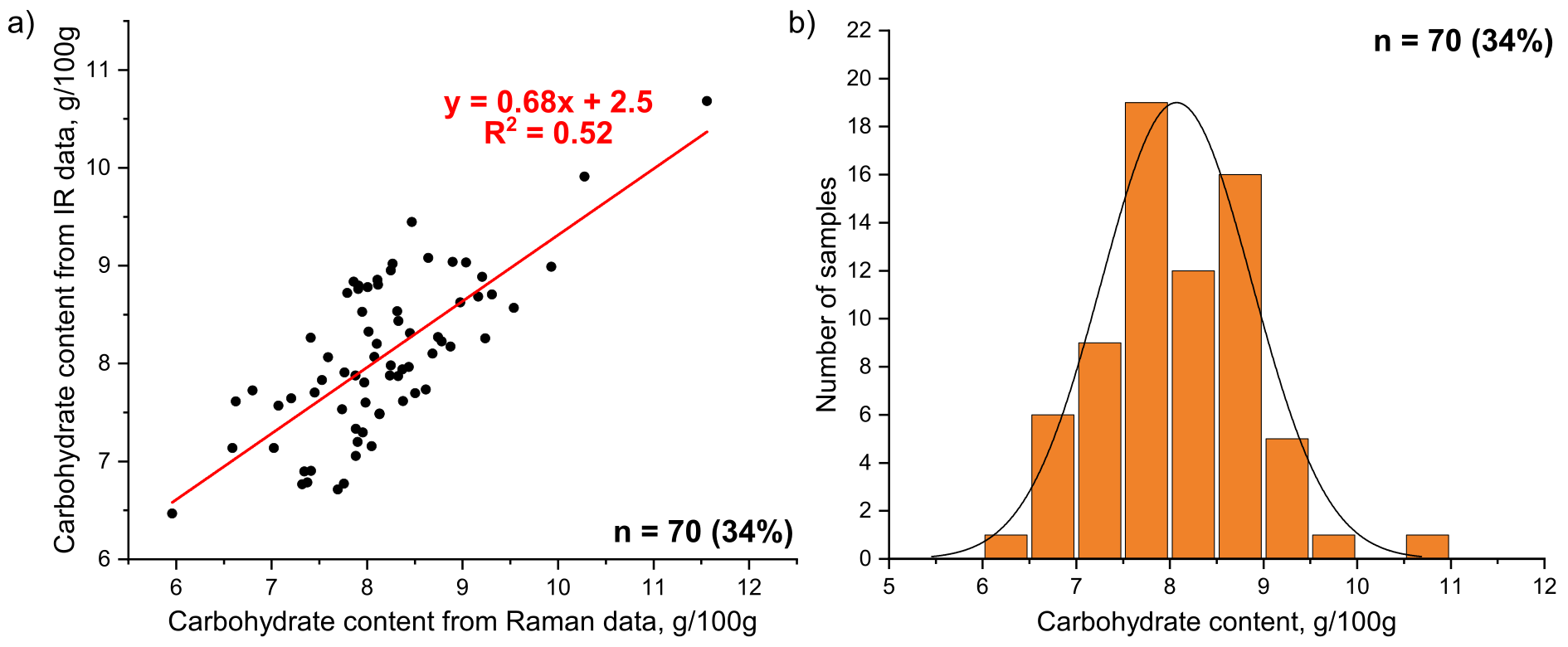


Fig. S19. (a) Comparison of the average determined carbohydrate content values from the filtered Raman and ATR-FTIR data. (b) Histogram of the determined average carbohydrate values from the respective PLS regression model using filtered ATR-FTIR spectroscopic data.
